# Supplementary material for: Heartwood Extract of Pterocarpus marsupium Roxb. Offers Defense against Oxyradicals and Improves Glucose Uptake in HepG2 Cells
Source: Metabolites. 2022 Oct 5;12(10):947. doi: 10.3390/metabo12100947 (PMC9607431; doi:10.3390/metabo12100947)
Supplement: Supplementary file 1 [file metabolites-12-00947-s001.zip › metabolites-1926590-supplementary.pdf]

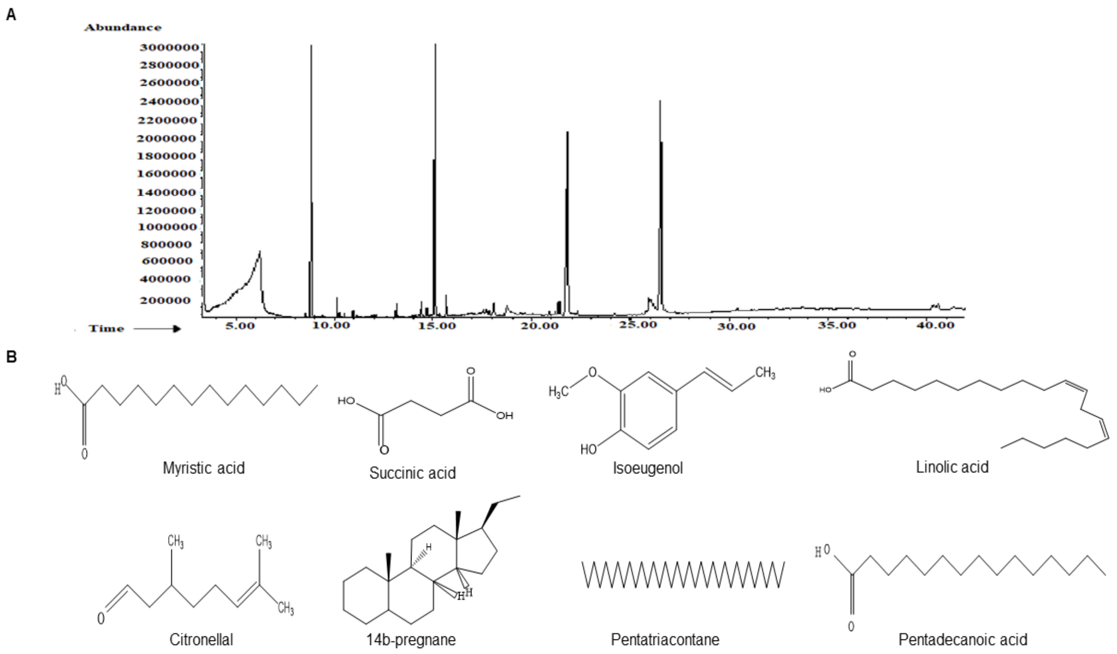

Figure S1. (A) GCMS chromatogram of MPME. (B) Chemical structure of 8 reported antidiabetic phytocompounds identified based on the retention time and peak area, namely Myristic acid (18.095 retention time and 0.80% peak area), Succinic acid (10.153 retention time and 0.76% peak area), (18.095 retention time and 0.80% peak area), Isoeugenol (14.691 retention time and 0.80% peak area), Pentadecanoic acid (19.658 retention time and 0.36% peak area), Oleic Acid (30.449 retention time and 0.19 % peak area), Citronellal (20.361 retention time and 0.36% peak area), *Pentatriacontane* (19.846 retention time and 0.02% peak area), 14b-pregnane (24.064 retention time and 0.02% peak area).

Table S1. Physiochemical parameters of MPME.

S.no.	Parameters	Percentage (%)
1	Total ash value	8
2	Acid insoluble ash	1.44
3	Water-soluble ash	1.36
4	Moisture content	1.25

Table S2. HPTLC fingerprint of MPME at 254 nm and 366 nm.

**S.No.**	**R_f**	**MPME**	
		**254 nm**	**366 nm**
1.	0.04	+	-
2.	0.24	+	+
3.	0.40	+	+
4.	0.44	+	+
5.	0.47	+	+
6.	0.51	+	-
7.	0.54	+	+
8.	0.57	+	-
9.	0.66	+	-
10.	0.77	+	+
11.	0.87	+	-
**Total number of metabolites**		**11**	**06**

Table S3. Linear regression data for calibration plots of the analyzed marker using the proposed HPTLC method.

Marker	Quercetin
Solvent system	Toluene: ethylacetate: formic acid: methanol (6:3:0.5:0.5)
Wavelength	254 nm
Retention factor (R_f)	0.47± 0.002
Linearity range (ng/spot)	200-4000
Regression equation	Y=1.066x + 458.5
Regression coefficient ± SD	0.993±0.011
Slope	1.066
LOD (ng/spot)	13.65
LOQ (ng/spot)	41.39
**Precision (% RSD)**	
Intra-day	0.44-0.79
Inter-day	1.44-1.66

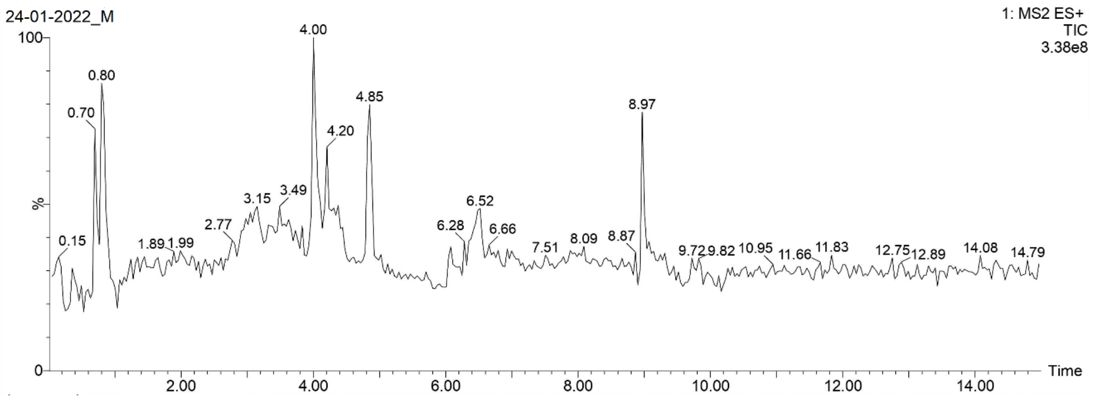

Figure S2. Ultra-performance liquid chromatography-mass spectroscopy (UPLC-MS) chromatogram.
